# Correction to: Downregulation of lncRNA ZNF582-AS1 due to DNA hypermethylation promotes clear cell renal cell carcinoma growth and metastasis by regulating the N(6)-methyladenosine modification of MT-RNR1

**DOI:** 10.1186/s13046-021-01969-9

**Published:** 2021-05-13

**Authors:** Wuping Yang, Kenan Zhang, Lei Li, Yawei Xu, Kaifang Ma, Haibiao Xie, Jingcheng Zhou, Lin Cai, Yanqing Gong, Kan Gong

**Affiliations:** 1 Department of Urology, Peking University First Hospital, No. 8, Xishiku Street, Xicheng District, Beijing, 100034 China; 2 Hereditary Kidney Cancer Research Center, Peking University First Hospital, No. 8, Xishiku Street, Xicheng District, Beijing, 100034 China; 3 Institute of Urology, Peking University, Beijing, 100034 People’s Republic of China; 4 National Urological Cancer Center, Beijing, 100034 People’s Republic of China

**Correction to: J Exp Clin Cancer Res 40, 92 (2021)**

**https://doi.org/10.1186/s13046-021-01889-8**

Following publication of the original article [1], the authors identified a minor error in image-typesetting in Fig. 4, specifically:

- Figure 4j: the first mouse lung of ZNF582-AS1 OE group has been replaced with the correct image

The corrected figure is given below. The correction does not have any effect on the results or conclusions of the paper. The original article has been corrected.

**Fig. 4:**
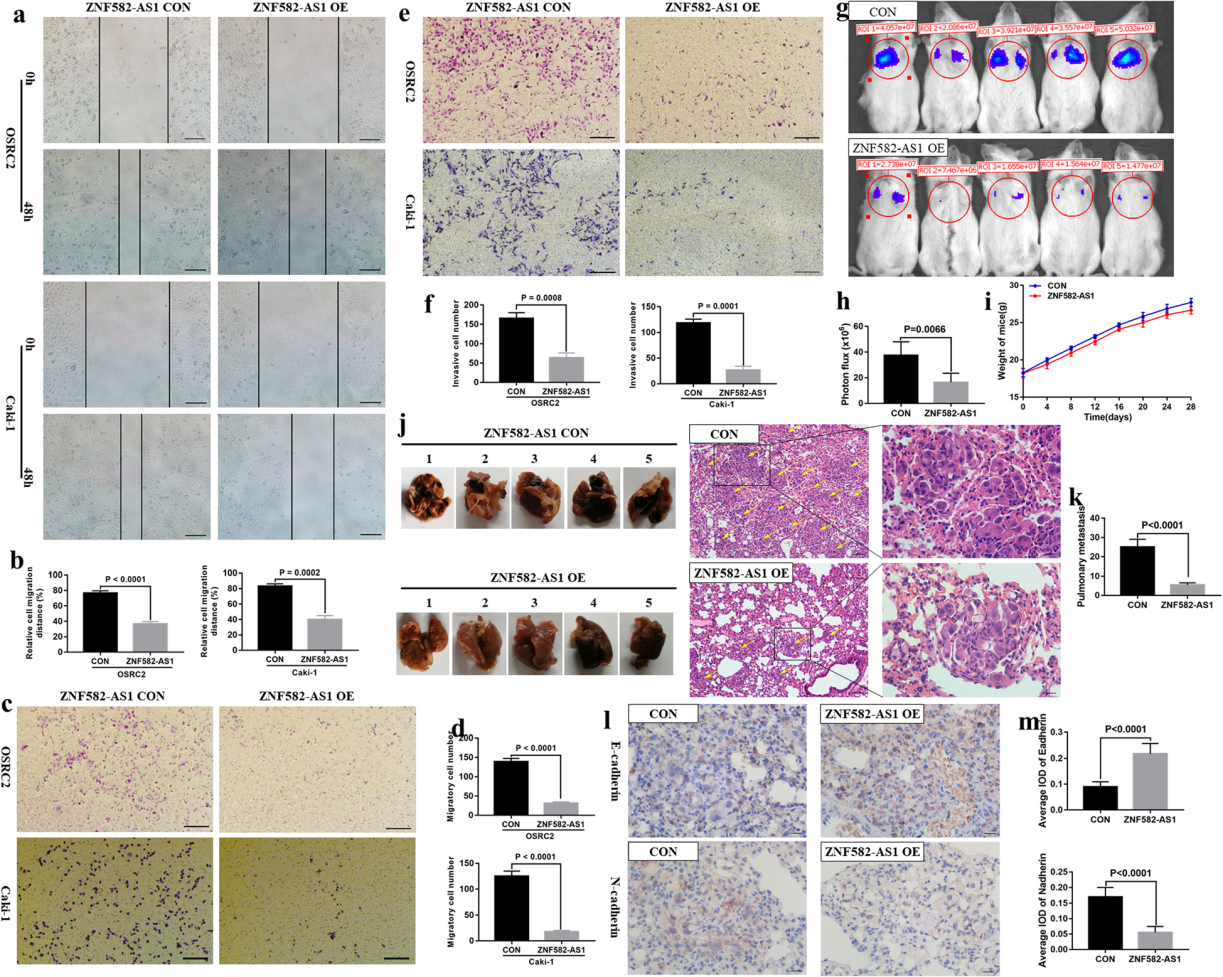
ZNF582-AS1 overexpression inhibited cell migratory and invasive ability in vitro and in vivo. **a** and **b** Wound healing assay determined the migratory distances of ZNF582-AS1-overexpressed OSRC2 and Caki-1 cells and their control cells. **c** and **d** Transwell migration and **e** and **f** invasion assays determined the migratory and invasive abilities of ZNF582-AS1-overexpressed OSRC2 and Caki-1 cells and their control cells. **g** and **h** The luciferase signals in the ZNF582-AS1-overexpressed group were remarkably lower than those in the control group. **i** There was no significant difference between the mice weight of ZNF582-AS1-overexpressed and control group. **j** and **k** The number and size of pulmonary metastases in the ZNF582-AS1-overexpressed group were significantly reduced compared with those in the control group. **l** and **m** ZNF582-AS1 overexpression increased Ecadherin expression and decreased N-cadherin expression in pulmonary metastases
